# Arteriovenous anastomosis learning curve using low cost simulator

**DOI:** 10.1590/1677-5449.190144

**Published:** 2020-11-11

**Authors:** Jéssika da Silva Antas, Ana Karolina Gama de Holanda, Achilles de Sousa Andrade, Alinne Mirlania Sabino de Araujo, Isabella Guilherme de Carvalho Costa, Luciano Ribeiro Dantas, Silvane Katerine Medeiros de Lima, Priscilla Lopes da Fonseca Abrantes Sarmento

**Affiliations:** 1 Universidade Federal da Paraíba – UFPB, Faculdade de Medicina, Departamento de Cirurgia, João Pessoa, PB, Brasil.

**Keywords:** learning curve, arteriovenous anastomosis, simulation training

## Abstract

**Background:**

In order to reduce difficulties with learning surgical techniques, supplementary tools for training were developed. This paper describes the learning curve followed by student volunteer research subjects who used an alternative model for practicing vascular anastomosis.

**Objectives:**

To evaluate the vascular anastomosis technique learning curve and development of manual skills using a low-cost experimental model.

**Methods:**

Experimental and prospective study using end-to-side vascular anastomosis in latex balloons over five successive phases, initiated after theoretical and practical guidance given by experienced vascular surgeon. The study subjects were six undergraduate medical students from Universidade Federal da Paraíba (UFPB), João Pessoa, PB, Brazil, in their third to fifth years of the course. Cluster analysis was used to interpret the data collected on the quality of anastomoses and the time taken.

**Results:**

The time taken to perform anastomosis reduced for all students, with statistical differences from phase 1 compared to phases 4 and 5. There was also a trend to increasing scores on the quality index as the phases progressed. However, no statistical differences were detected using the Friedman test, which is appropriate for data measured with ordinal levels (quality was assessed on a scale of 1 to 5).

**Conclusions:**

It was found that the training model used was effective for increasing learning of this technique. It is believed that future studies with larger samples or a higher number of phases could demonstrate both reduced time and improved quality of the anastomoses performed with statistical significance.

## INTRODUCTION

Teaching surgical techniques remains a challenge at medical schools, since, particularly in this area of medicine, practical and manual skills cannot be perfected with theoretical teaching alone.^1^ One option for more effective teaching would be to use simulators or in vivo animal models.^2^ However, uses of these practices is beset by the high cost of simulators and ethical issues with regard to use of animals for training, which are subject to much debate currently.^3^

Vascular anastomosis techniques are widely employed to create arteriovenous fistulas for hemodialysis or for vascular bypass surgery, but training in these techniques is very often neglected during undergraduate courses and even during residency in surgical areas because of ethical issues and financial limitations. As a consequence, in our country and in some other settings, the physician’s first practical experience with anastomoses will be with a patient undergoing vascular surgery.^4^ However, we do not believe that it is acceptable that human beings be used as tools for training.

Although the lack of ex vivo training means that initial training in surgery in vivo is the only option at many centers, this has the consequence of increasing the duration of surgery, which, in turn, is considered an independent risk factor for surgical complications.^5^^-^9 It has already been reported that the time taken to perform vascular anastomoses is longer among residents and students. However, training with animal models and simulators results in improved speed and technical refinement, which is reflected in improvements when surgical procedures are conducted.^10^^-^12

This being so, a number of alternative models have been developed for teaching and development of vascular anastomosis skills, employing a variety of materials ranging from the synthetic, such as rubber and silicone gloves, to vegetable produce.^13^^,^^14^ One such model, which was used in this study, was created using latex balloons^15^ and offers the advantages of low cost, simple construction, the possibility of re-use, and similar diameter and consistency to vascular structures typically involved in procedures in vivo, such as brachiocephalic fistulas and femoropopliteal bypass.

The objective of this study was to evaluate the vascular anastomosis learning curve and development of manual skills when using an experimental, low cost model. The analysis of students’ progress in learning the vascular anastomosis construction technique is based on reduction of the time each student takes to perform an anastomosis and improvements in the quality of their technique over the course of the study, using each student’s initial performance as their own control. In this study, as in surgical practice, neither a poor quality anastomosis performed rapidly nor an anastomosis of excellent quality that takes a very long time are desirable.

## METHODS

This is a prospective experimental study that was submitted for analysis by the Research Ethics Committee at the Universidade Federal da Paraíba (UFPB), João Pessoa, PB, Brazil, and approved under protocol number 96134418.6.0000.8069. The project complies with resolution 466 of December 2012, which relates to respect for human dignity and due protection for all research participants. All of the volunteers involved signed individual consent forms. Planning and data collection occurred from September 2018 to June 2019. The experimental subjects were six undergraduate students on the sixth to tenth semesters of the Medicine degree at UFPB. At the time of the study, these students were monitors on the Foundations of Surgical Technique module. The students who participated had no prior technical knowledge of vascular anastomoses, which was one of the reasons for conducting this project. They did have basic knowledge of surgery gained from the theoretical and practical content of the Foundations of Surgical Technique module, such as surgical instruments, surgical threads, and the principals of surgical sutures with continuous and interrupted stitches. This module is taken during the fourth semester of the UFPB Medicine degree. The sample size used in this observational study was not calculated statistically, which could constitute a limitation to the results observed.

**Figure 1:**
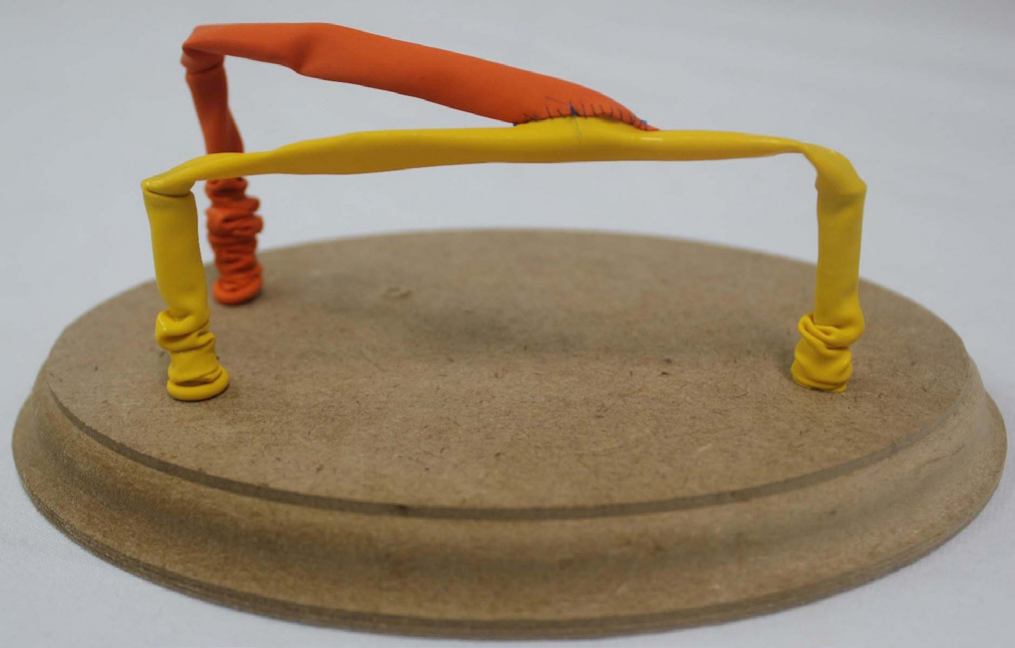
Prototype constructed with wood, screws, and latex balloons used to perform anastomoses.

**Figure 2:**
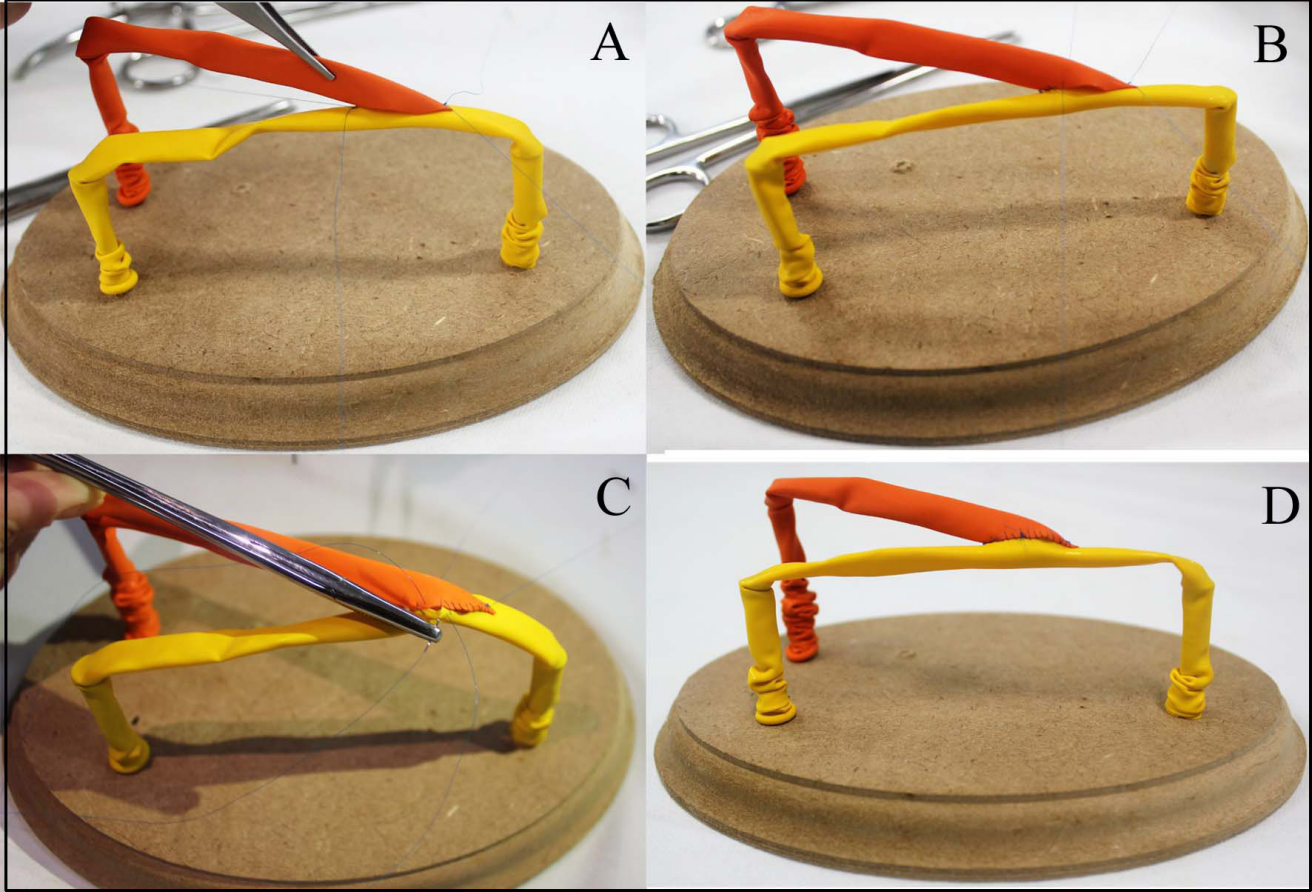
Construction of end-to-side anastomosis: (A) Initial suture approximating the balloons with sutures at the angles (proximal and distal); (B) continuous sutures along four quadrants; (C) detail of needles passing entirely through the wall of the balloon; (D) final appearance of the anastomosis.

The end-to-side anastomosis technique was chosen for teaching and analysis in this study. Initially, the student participants were given practical and individual instruction by an experienced vascular surgery professor, based on a demonstration of performing an end-to-side anastomosis using the technique recommended by Rutherford,^16^ with initial sutures at the angles and closure of the anterior and posterior walls with continuous sutures.^16^^,^^17^ The end-to-side anastomosis was performed on the alternative low cost training model, constructed with a wooden board, screws, and latex balloons^15^ (Figure 1), and a surgical materials kit containing four straight forceps for sutures, two needle holders, one pair of scissors, and 5-0 polypropylene suture material, which were used to conduct the vascular anastomosis. The size of the longitudinal incision in the recipient balloon (side vessel) was more than one and a half times the caliber of the balloon, and the end of the balloon to be anastomosed was cut at an angle of 30 to 45 degrees. Some of the steps involved in constructing the end-to-side anastomosis^15^ are illustrated in Figure 2.

The students were then divided into three pairs to perform anastomoses, with one student in each pair acting as surgeon and the other as surgical assistant and vice-versa. Thus, each student conducted one anastomosis supervised by the surgery professor and assisted by the other student, which was filmed to enable them to memorize the technique more easily. This first anastomosis attempt was not computed in the statistical analysis of the study, since it was part of teaching the technique.

The latex balloons representing the artery and vein had a diameter of 1 cm and the orifice of the balloon representing the artery was 2 cm in length. The students only performed suturing of the balloons. The balloon representing the vein was cut to the ideal proportional size for vascular anastomosis. All of the students were instructed not to practice the technique other than during the scheduled sessions.

Each student performed five vascular anastomoses in succession, with a 1-week interval between each attempting, making a total of five phases (1, 2, 3, 4, and 5). The pairs were not changed over the course of the experiment. The assistant was responsible for timing the time taken to perform each vascular anastomosis and for noting any intercurrent conditions if any occurred. Each student was identified by a letter (A, B, C, D, E, or F) and each anastomosis was identified by the student’s letter and the phase in which it was performed – for example, A2: student A, phase 2. At the end of each attempt, the anastomosis was put into a sealed envelope for evaluation by two surgeons, independently, who rated them using a scale created for the activity, scored from 0 to 5. The scale comprised the following questions: a) Were the sutures equally spaced properly? b) Were the sutures the correct distance from the edge of the vessel? c) Were the sutures correctly tensioned? d) Were the edges of the suture free from inversion? and e) Was the procedure performed without complications? Each question was answered “yes” or “no”. One point was scored for each question answered “yes”.

## RESULTS

**Figure 3:**
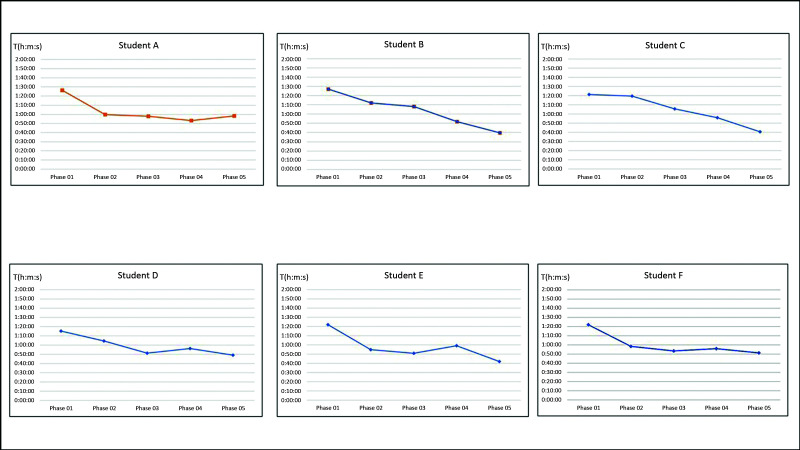
Curve showing change in the time taken by students to perform anastomoses against study phases. Time (T) in hours, minutes, and seconds (h:m:s).

The data on the time taken to perform anastomosis relate to five repetitions per student, repeating the same procedure in each of the five phases. Figure 3 shows the curves of change in anastomosis time over the five study phases for each student.

**Figure 4:**
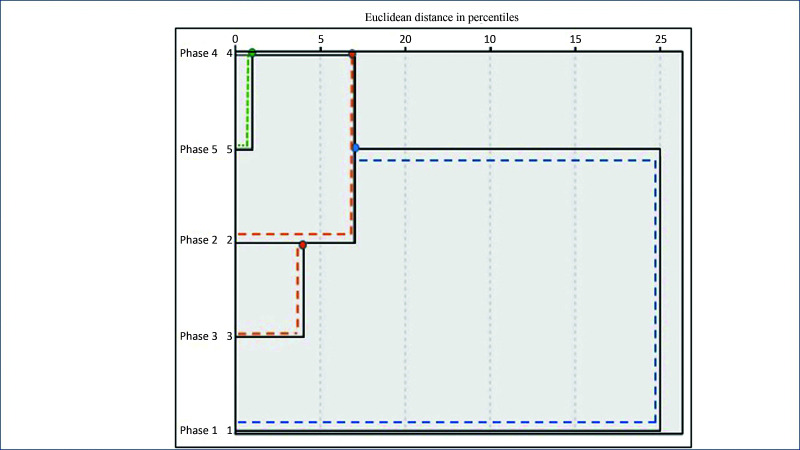
Dendrogram showing clusters by Euclidean distances in percentiles for times observed in phases 1 to 5.

Cluster analysis was used to evaluate difference in time gained per phase, using the agglomerative hierarchical clustering method with Euclidean distances. This analysis yields a tree diagram, known as a dendrogram, which is shown in Figure 4.

According to this dendrogram, phase 1 (area outlined in blue) was different from the other phases, i.e. with a longer time than the other phases. Therefore, the time taken in phases 2 and 3 (areas outlined in orange) was shorter than phase 1 and longer than phases 4 and 5 (areas outlined in green), delineating three clusters (C_1_ = phase 1; C_2_ = phases 2 and 3; and C_3_ = phases 4 and 5), based on the distribution of anastomosis times.

The x-axis of Figure 4 is marked in values at intervals of 5, with the values 0, 5, 10, 15, 20, and 25, which are the Euclidean distances, i.e., they indicate the proximity of each record to the others. It can be observed how far phase 1 is from the other phases. Additionally, we can also observe that phase 2 is between percentiles 5 and 10; whereas phase 3 is below the 5th percentile, showing that the time taken to perform anastomosis was already well below the time taken in phases 1 and 2. However, observing phases 4 and 5, it can be seen that they are very close to percentile 0, showing that there is no significant difference between these two phases in terms of the relationship between times taken to perform anastomoses. This is a positive cluster analysis result, demonstrating that there was indeed a reduction in the time taken to perform anastomoses and that by phase 4 the students had reached a plateau in terms of the time required.

If the dendrogram is analyzed using a vertical cutoff through the tree, we can observe that the time for C_1_ = phase 1 was infinitely greater than for the other phases. With a cutoff at C_2_ = phases 2 and 3, we observe a minimal difference between the time taken to perform anastomoses in phases 2 and 3. In C_3_ = phases 4 and 5, the cluster analysis shows there was no significant difference between the anastomosis times for phases 4 and 5. This evidence suggests that if the experiment had been terminated after phase 4, there would have been no impact on the analysis of anastomosis times.

Both mean and median times reduced as the phases progressed (as shown in Table 1) and comparison with analysis of variance (ANOVA) for repeated measures yielded p values > 0.05 for all phases, since the phases data were normally distributed according to the Shapiro-Wilk test. The F test p value is 0.001, providing statistical evidence that the mean phase times are different. This evidence is confirmed by the cluster analysis shown Figure 4.

**Table 1:** Descriptive measures of phases and repeated measures comparison of change in time taken.

**Phase**	**Mean**	**SD**	**Median**	**p**
1	4939.0	260.53	4917.50	0.001
2	3890.0	557.14	3718.00	
3	3193.6	829.85	3146.00	
4	3326.6	147.49	3357.00	
5	2805.1	442.66	2736.50	

SD: standard deviation.

**Figure 5:**
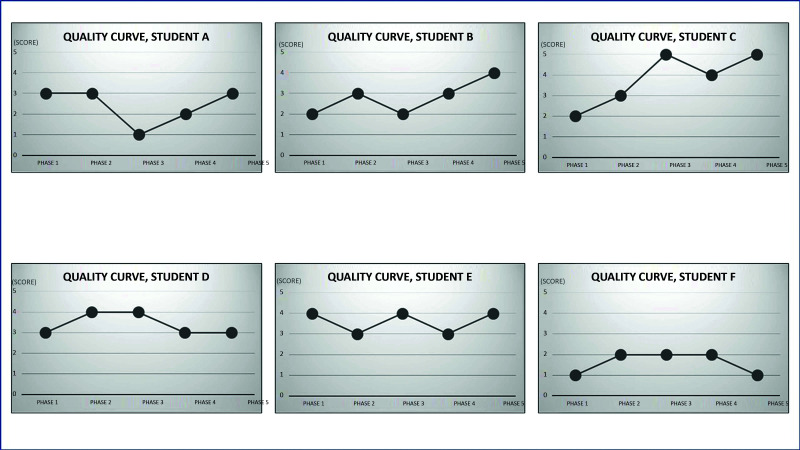
Curves illustrating quality of anastomoses (score) for each student over the course of the five study phases.

**Figure 6:**
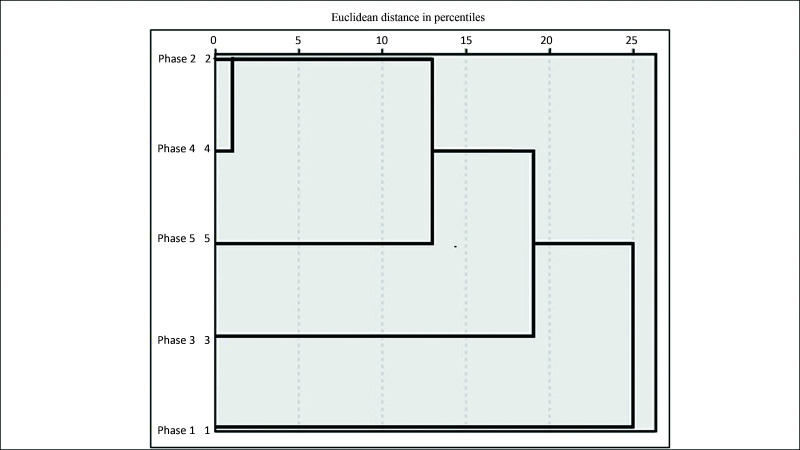
Dendrogram of hierarchical method clusters by Euclidean distances in percentiles for quality scores by phase.

Figure 5 illustrates the change in anastomosis quality scores for each student over the course of the study. For analysis of the quality scores, a dendrogram constructed using the same clustering method as before is shown in Figure 6. In the same manner as in Figure 4, it can be observed that the dendrogram forms four cluster at a vertical line corresponding to a distance equal to 5: C_1_ = phase 1, C_2_ = phase 3, C_3_ = phase 5, and C_4_ = phases 2 and 4. Phase 5 has the highest quality score, showing that the students had achieved their highest level of quality.

**Figure 7:**
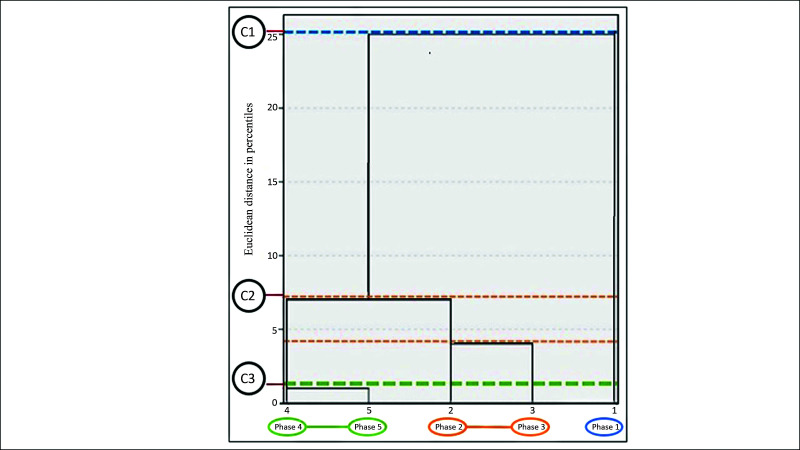
Dendrogram showing clusters by Euclidean distances in percentiles for times observed in phases 1 to 5 (rotated 90° counterclockwise).

If the dendrogram shown in Figure 4 is rotated through 90° counterclockwise, horizontal cutoffs illustrated by the lines (blue, orange, and green) are plotted at the levels corresponding to the time taken in each phase, as demonstrated in Figure 7. We can thus observe that the time taken in C1 (phase 1) was considerably greater than the time taken in the other phases. With a cutoff at C_2_ (phases 2 and 3), we observe a minimal difference between the time taken to perform anastomoses in phases 2 and 3. In C_3_ (horizontal phase 4 and 5), the cluster analysis shows there was no significant difference between the anastomosis times for phases 4 and 5. This evidence suggests that if the experiment had been terminated after phase 4, there would have been no impact on the analysis of anastomosis times

In Table 2, it can be observed that there was a trend for the quality index to increase as the phases progressed and that there was no statistical difference using the Friedman test, which is appropriate for data measured with ordinal levels (quality assessed on a scale of 1 to 5). Therefore, the statistical method does not detect a difference, but clinical differences are revealed by the median statistical measure and the cluster analysis. It is probable that a larger sample size would detect a statistical difference between phases using the Friedman hypothesis test.

**Table 2:** Descriptive measures of quality index scores and comparison over the phases.

**Phase**	**Mean**	**SD**	**Median**	**p**
1	2.50	1.049	2.50	0.603
2	3.00	0.632	3.00	
3	3.00	1.549	3.00	
4	2.83	0.753	3.00	
5	3.33	1.366	3.50	

SD: standard deviation.

## DISCUSSION

Surgical technique can be defined as a set of manual or instrumented maneuvers performed by the surgical team to accomplish an operation. Thus, the knowledge underlying surgical technique includes handling of instruments and general manipulation of tissues used to execute maneuvers common to all surgical procedures.^17^^-^18 Development of manual skills demands continuous practical training starting during the undergraduate course and continuing through medical residency.

Vascular anastomoses are not only important in vascular surgery, but also for surgical practice in general, such as for myocardial and cerebral revascularization, in trauma care, transplantations, plastic surgery, and to repair iatrogenic injuries, making them an essential part of training in medical residency programs.^19^^-^21 Traditionally, residents acquired technical skills in the operating room, under supervision by a treating surgeon. However, over recent years a number of studies have presented proposals for training to give future surgeons experience with vascular anastomoses before residency.^4^

Of these, animal models are frequently cited in the literature, offering the advantage of proximity to the physiological, anatomic, and organic characteristics of human beings.^22^ Achar et al.^2^ developed a model using chicken trachea and esophagus for training end-to-end anastomoses. Garbin et al.^23^ compared students’ learning after training side-to-side arteriovenous anastomosis with a model using bovine tongue. However, in addition to the ethical issues, this type of model also requires an appropriate space for preparing and storing the materials used.

In contrast, the medical industry has developed artificial simulators for training vascular anastomoses, but their elevated cost is a barrier to access for a large proportion of Brazilian institutions. This has led to development of alternative simulators as proposals for more accessible training models.^13^^-^15 In our study, the alternative training model was chosen because of unavailability of access to animal models or the simulators available on the market. Thus, in line with the conditions at our institution, a model using inert materials that is reproducible and low cost was the most appropriate choice.

It is known that simulation with inanimate models offers the possibility of repetitive training, leading to improved performance, ensuring the student security and no possibility of causing harm to patients.^23^^-^25 Atlan et al.^26^ developed a synthetic model for training microvascular anastomoses using gelatin tubes with polyvinyl alcohol and compared it with vessels from rodents, demonstrating improved performance and reduced use of animals.

The simulator proposed by Sarmento et al.^15^ proved viable and low cost but had not yet been tested with undergraduate students. According to that study, practicing anastomoses using this simulator enabled familiarization with instruments specific to vascular surgery and with use of fine caliber suture thread and two needles, improving dexterity and agility in a technique employing delicate movements. From this perspective, we evaluated the model developed using learning curves.

Learning curves are widely used because they enable evaluation of an individual’s progress over the course of several repetitions of a given technique. In general, the parameters most used are time taken and quality of the procedure.^27^ A study with radiology residents using training with simulation of joint punctures demonstrated improvements in time and quality.^28^ Yoshida et al. also described reductions in time taken in a study conducted with vascular surgeons.^11^

It is important to emphasize that reduction in the time taken to perform a procedure is not in itself enough to conclude that a given skill is being improved. It is also necessary to observe improvement in technical parameters that predict the quality of the procedure, in order to reduce perioperative complications. A study with general surgery residents related competence in a laboratory model to surgical competence, analyzing not only the time taken to conclude anastomosis, but also the degree of anastomotic leakage and the number of leaks.^29^

In the literature, the most cited score for assessing vascular anastomosis performance is the Objective Structured Assessment of Technical Skills (OSATS), which can be used to assess surgical skills in a reliable and valid manner. This method is based on direct observation of residents executing a variety of structured tasks.^30^ However, in view of the need for either an examiner or for recording all of the phases, it was decided to employ a personalized score that could be used to conduct assessments after each anastomosis had been completed.

In the present study, it was observed that the time taken by all of the students reduced, with statistical differences between phase 1 and phases 4 and 5. The descriptive analysis revealed a trend to increasing quality of the anastomoses as the phases progressed, in terms of clinical differences in the median statistical measure and in the cluster analysis.

However, no statistically significant difference was detected over the course of the phases. This might be because it is necessary to perform many repetitions to achieve good quality. A study with 15 surgeons who performed coronary surgery demonstrated that 4 years of experience are needed to achieve technical competence and that the total training time progressively reduces mortality.^31^ It is therefore probable that a study with a larger number of phases or a sample with a larger number of students would detect statistically significant improvements in quality.

Notwithstanding, it is also important to point out that there is great variation in students’ manual skills. It is known that learning of surgical skills is influenced by a complex interaction between factors that include the person’s innate capability, prior surgical experience, and motivation.^32^ Considerable interindividual variability was observed in a study comparing learning curves of videolaparoscopy students: while some students improved gradually, others made abrupt improvements.^33^ Some people will therefore find it easier to learn a given procedure than others, which also changes the time needed for each student to achieve the necessary quality. Moreover, achieving perfect quality was not the primary objective our study, which was designed to aid in learning the surgical technique.

Thus, the alternative, low-cost model using latex balloons introduced students of the subject to the vascular anastomosis technique and was an effective tool for improving overall learning of the technique. As future prospects, we hope to include this prototype in medical residency programs as a means of helping to train residents and, in turn, reduce the need to practice on patients.
