# Supplementary material for: Arteriovenous anastomosis learning curve using low cost simulator
Source: J Vasc Bras. 2020 Nov 11;19:e20190144. doi: 10.1590/1677-5449.190144 (PMC8218093; doi:10.1590/1677-5449.190144)
Supplement: Translation

# Translation: Curva de aprendizado de anastomose arteriovenosa com uso de simulador de baixo custo

**Authors:** Jéssika da Silva Antas, Ana Karolina Gama de Holanda, Achilles de Sousa Andrade, Alinne Mirlania Sabino de Araujo, Isabella Guilherme de Carvalho Costa, Luciano Ribeiro Dantas, Silvane Katerine Medeiros de Lima, Priscilla Lopes da Fonseca Abrantes Sarmento

**Affiliations:** 1 Universidade Federal da Paraíba – UFPB, Faculdade de Medicina, Departamento de Cirurgia, João Pessoa, PB, Brasil.

**Keywords:** curva de aprendizado, anastomose arteriovenosa, treinamento por simulação

## Abstract

**Contexto:**

A redução das dificuldades no aprendizado da técnica cirúrgica levou ao surgimento de ferramentas complementares. Este trabalho descreve a curva de aprendizado dos alunos sujeitos da pesquisa, utilizando um modelo alternativo para a prática de anastomose vascular.

**Objetivos:**

Avaliar a curva de aprendizado da técnica de anastomose vascular e do desenvolvimento de habilidades manuais a partir da utilização de um modelo experimental de baixo custo.

**Métodos:**

Este trabalho é um estudo experimental e prospectivo, com a realização de anastomoses vasculares do tipo terminolateral em balões de látex durante cinco fases sucessivas, iniciadas após orientação teórico-prática de uma cirurgiã vascular experiente. Os sujeitos foram seis graduandos do curso de Medicina, do terceiro ao quinto ano, da Universidade Federal da Paraíba (UFPB), João Pessoa, Paraíba, Brasil. A interpretação do material coletado acerca da qualidade das anastomoses e do tempo utilizado seguiu a análise de agrupamento.

**Resultados:**

Houve redução do tempo de realização das anastomoses de todos os alunos, com diferença estatística da fase 1 quando comparada às fases 4 e 5, bem como tendência crescente no índice de qualidade ao longo das fases. Porém, não foi detectada diferença estatística a partir do teste de Friedman, apropriado para dados com nível de mensuração ordinal (escala de 1 a 5 na avaliação da qualidade).

**Conclusões:**

O modelo de treinamento utilizado foi efetivo para incremento do aprendizado dessa técnica, acreditando-se que amostras de maior tamanho ou com maior número de fases em trabalhos futuros poderiam demonstrar redução do tempo associada a melhora da qualidade da anastomose realizada com significância estatística.

## INTRODUÇÃO

O ensino da técnica cirúrgica continua sendo um desafio nas instituições de ensino médico, uma vez que, particularmente nessa área da medicina, as habilidades práticas e manuais não são aperfeiçoadas apenas com o ensino teórico^1^. Uma alternativa para o aprendizado mais efetivo seria a utilização de simuladores ou de modelos animais *in vivo* 2. Entretanto, essas práticas encontram como obstáculo o elevado custo dos simuladores e as questões éticas, muito discutidas atualmente acerca da utilização de animais para treinamento^3^.

As técnicas de anastomose vascular são comumente utilizadas para a confecção de fístulas arteriovenosas para hemodiálise ou para cirurgias de *bypass* vascular, porém o ensino dessas técnicas é muitas vezes preterido durante a graduação e até mesmo durante a residência das áreas cirúrgicas devido a limitações éticas e financeiras. Como consequência, na nossa realidade e em alguns locais, o primeiro contato prático do médico com as anastomoses acontece no caso de paciente submetido a cirurgia vascular^4^. Contudo, não concordamos que o ser humano seja uma ferramenta de aprendizado.

A falta de treinamento *ex vivo* e o início de treinamento na cirurgia *in vivo*, apesar de ser a única alternativa em muitos centros, têm como consequência o aumento do tempo cirúrgico, que, por sua vez, é considerado um fator de risco independente para complicações cirúrgicas^5^^-^9. Já foi descrito que o tempo para a realização de anastomoses vasculares é maior entre residentes e estudantes. Por outro lado, através do treinamento em modelos animais e simuladores, ocorre uma melhora no tempo e no aperfeiçoamento da técnica, que reflete na melhora no decorrer da realização dos procedimentos cirúrgicos^10^^-^12.

Assim sendo, alguns modelos alternativos para o aprendizado e desenvolvimento de habilidades em anastomose vascular foram desenvolvidos, utilizando desde materiais sintéticos, como luvas de borracha e silicone, até produtos vegetais^13^^,^^14^. Um desses modelos, utilizado neste estudo, foi criado a partir de balões de látex^15^, apresentando como vantagem o baixo custo, a facilidade de confecção, a possibilidade de reutilização, bem como o diâmetro e a consistência semelhantes a estruturas vasculares tipicamente utilizadas em procedimentos *in vivo*, como fístulas braquiocefálicas e *bypass* femoropoplíteo.

O objetivo deste estudo foi avaliar a curva de aprendizado da técnica de anastomose vascular e do desenvolvimento de habilidades manuais a partir da utilização de um modelo experimental de baixo custo. A avaliação do aprendizado na confecção da anastomose vascular analisa a diminuição do tempo gasto para a execução da anastomose acompanhado pela melhora da qualidade da técnica de cada estudante no decorrer do estudo, sendo o desempenho inicial de cada estudante o seu próprio controle. Neste estudo, assim como na prática cirúrgica, não são desejáveis uma anastomose realizada de maneira rápida com qualidade ruim nem uma anastomose de ótima qualidade, porém com tempo de execução muito prolongado.

## MÉTODOS

Trata-se de um estudo experimental e prospectivo, submetido ao Comitê de Ética em Pesquisa da Universidade Federal da Paraíba (UFPB), João Pessoa, PB, Brasil, e aprovado sob número 96134418.6.0000.8069. Este projeto está de acordo com a resolução n° 466 de dezembro de 2012, que considera o respeito pela dignidade humana e pela devida proteção a todos os participantes da pesquisa. Todos os sujeitos envolvidos assinaram um termo de consentimento individual. O planejamento e a coleta de dados foram realizados entre setembro de 2018 e junho de 2019. Os sujeitos do experimento foram de seis alunos, todos graduandos do curso de Medicina da UFPB entre o sexto e décimos períodos. Esses alunos, na ocasião do estudo, exerciam atividades de monitoria da disciplina de Bases da Técnica Cirúrgica. Os alunos participantes não tinham conhecimento técnico prévio sobre anastomoses vasculares, o que, inclusive, foi um motivo para o desenvolvimento deste projeto. Tinham conhecimento cirúrgico básico a partir do conteúdo teórico e prático das aulas da disciplina de Bases da Técnica Cirúrgica, como instrumentos em cirurgia, fios cirúrgicos e princípios de sutura cirúrgica com pontos contínuos e descontínuos. Essa disciplina é cursada no quarto período do curso de Medicina da UFPB. O tamanho da amostra utilizado neste estudo observacional não foi calculado de maneira estatística, podendo configurar como uma limitação para os resultados observados.

A anastomose terminolateral foi o tipo de técnica escolhido para o aprendizado e a análise neste estudo. Inicialmente, os discentes participantes foram orientados de maneira prática e individual por uma professora cirurgiã vascular experiente, a partir da demonstração com realização da anastomose terminolateral, conforme a técnica recomendada por Rutherford^16^, com pontos iniciais nos ângulos e fechamento das paredes anterior e posterior com sutura contínua^16^^,^^17^. A anastomose terminolateral foi realizada em modelo de treinamento alternativo de baixo custo, elaborado a partir de placa de madeira, parafusos e balões de látex^15^ (Figura 1), e um kit de material cirúrgico, que continha quatro pinças para reparo de fios, dois porta-agulhas, uma tesoura e fios polipropileno 5-0, foi utilizado para a realização da anastomose vascular. O tamanho da incisão longitudinal no balão receptor (lateral) foi maior que uma vez e meia o calibre do balão, e o segmento terminal do balão a ser anastomosado foi cortado com angulação de 30 a 45 graus. Algumas etapas da confecção da anastomose terminolateral^15^ são demonstradas na Figura 2.

**Figura 1:** Protótipo construído com madeira, parafusos e balões de látex utilizado para a realização das anastomoses.

**Figura 2:** Confecção da anastomose terminolateral: (A) sutura inicial aproximando os balões com pontos nos ângulos (proximal e distal); (B) sutura contínua em quatro quadrantes; (C) detalhe na passagem da agulha em toda a parede do balão; (D) aspecto final da anastomose.

Posteriormente, os estudantes foram divididos em três duplas para a confecção da anastomose, sendo um aluno posicionado como cirurgião e outro aluno, como auxiliar e vice-versa. Dessa maneira, cada discente realizou uma anastomose supervisionada pela professora cirurgiã e com auxílio de outro discente, a qual foi filmada para permitir melhor fixação da técnica. Essa primeira anastomose não foi computada para análise estatística do estudo, já que se tratava do ensino da técnica.

Os balões de látex representativos da artéria e veia possuíam 01 cm de diâmetro e o orifício do balão representativo da artéria possuía 02 cm de comprimento. Os discentes realizaram apenas a sutura dos balões. A bexiga representativa da veia foi cortada em tamanho proporcional ideal para a realização da anastomose vascular. Todos os alunos foram instruídos a não praticar a técnica fora das sessões predeterminadas.

Cada aluno prosseguiu com a realização de cinco anastomoses vasculares sucessivas com um intervalo de uma semana entre cada, totalizando cinco fases (1, 2, 3, 4 e 5). Não houve mudança de duplas durante o experimento. Dessa forma, o auxiliar ficou responsável por cronometrar e anotar o tempo de realização de cada anastomose vascular, bem como apontar intercorrências caso tenham ocorrido. Cada aluno foi identificado por uma letra (A, B, C, D, E ou F) e, em cada anastomose, foi anotada a letra correspondente ao aluno e à fase em que se encontrava, referentes à sequência de realização das anastomoses – por exemplo, A2: aluno A e fase 2. Ao final de cada performance, a anastomose foi colocada em um envelope lacrado para avaliação por dois cirurgiões, de forma independente, por meio de um escore criado para a atividade, com valor de 0 a 5. O escore continha as seguintes perguntas: a) Realizou pontos equidistantes de maneira adequada? b) Realizou pontos com distância adequada da borda do vaso? c) A sutura apresenta tensão adequada? d) As bordas da sutura estão sem inversão? e e) O procedimento foi realizado sem intercorrências? Essas questões foram respondidas com “sim” ou “não”. A cada resposta “sim”, um ponto foi atribuído.

## RESULTADOS

Os dados do tempo de execução das anastomoses foram realizados em cinco repetições para cada discente e, a cada uma das cinco fases, se repetia o mesmo procedimento. A Figura 3 demonstra a curva de evolução do tempo para a execução da anastomose de cada aluno durante as cinco fases do estudo.

**Figura 3:** Curva de evolução de tempo *versus* fases do estudo para a execução das anastomoses dos alunos participantes. Tempo (T) descrito em horas, minutos e segundos (h:m:s).

Para avaliar o ganho diferenciado por tempo nas fases, a análise de agrupamento foi aplicada, com o método hierárquico de ligação entre grupos com a distância euclidiana. A análise gerou um diagrama de árvore, chamado de dendrograma, apresentado na Figura 4.

**Figura 4:** Dendrograma entre grupos pela distância euclidiana em percentis referente aos tempos observados nas fases 1 a 5.

De acordo com esse dendrograma, a fase 1 (seguimento pontilhado em azul) apresentou-se diferenciada das demais fases, ou seja, apresentou um tempo de execução maior em relação às outras fases. Assim, o tempo de execução das fases 2 e 3 (seguimentos pontilhados em laranja) mostraram-se menores que o da fase 1 e maiores que os das fases 4 e 5 (seguimentos pontilhados em verde), evidenciando, assim, três agrupamentos (G_1_ = fase 1; G_2_ = fases 2 e 3; G_3_ = fases 4 e 5), a partir da distribuição dos tempos de duração das anastomoses.

No eixo x da Figura 4, observamos valores espaçados com as numerações 0, 5, 10, 15, 20, 25, que nada mais são do que a distância euclidiana, ou seja, o quão próximo um registro está de outro – podemos notar o quão distante a fase 1 está das demais fases. Além disso, notamos que a fase 2 encontra-se entre os percentis 5 e 10; já na fase 3 nota-se que ela está abaixo do percentil 5, o que quer dizer que o tempo de realização da anastomose já foi muito abaixo das fases 1 e 2. Porém, ao observar as fases 4 e 5, notamos que estão bem próximas do percentil 0, o que mostra que não há diferença significante entre essas duas fases em relação ao tempo de realização das anastomoses. Isso responde positivamente à análise de agrupamento, demonstrando que de fato há uma diminuição do tempo entre a realização das anastomoses e que já na fase 4 os indivíduos atingem um platô em relação ao tempo.

Quando o dendrograma é analisado através de um corte vertical na árvore, observamos que o G_1_ = fase 1 apresentou um tempo infinitamente maior que as demais fases. No corte em G_2_ = fases 2 e 3, observamos uma diferença mínima entre o tempo de realização das anastomoses das fases 2 e 3. Já no G_3_ = fases 4 e 5, pela análise de agrupamentos, não há diferença significativa entre o tempo das anastomoses das fases 4 e 5. Essas evidências sugerem, assim, que, se o experimento fosse finalizado na fase 4, não haveria prejuízo na análise do tempo de realização das anastomoses.

As médias e medianas diminuíram de acordo com a progressão das fases (como pode ser visto na Tabela 1), e sua comparação com a análise de variância (ANOVA) com medidas repetidas apresentaram valor de p > 0,05 em todas as fases, uma vez que os dados das fases apresentam distribuição normal segundo o teste de Shapiro-Wilk. O valor de p pelo teste F é 0,001, fornecendo evidência estatística de que as fases apresentam tempos médios diferentes. Essa evidência foi confirmada com a análise de agrupamento apresentada na Figura 4.

**Tabela 1:** Medidas descritivas das fases e sua comparação com a variação do tempo para medidas repetidas.

**Fase**	**Média**	**DP**	**Mediana**	**Valor - p**
1	4939,0	260,53	4917,50	0,001
2	3890,0	557,14	3718,00	
3	3193,6	829,85	3146,00	
4	3326,6	147,49	3357,00	
5	2805,1	442,66	2736,50	

DP: desvio-padrão.

Na Figura 5, podemos observar a curva de evolução do escore de qualidade da anastomose de cada aluno durante o estudo. Para a análise do escore de qualidade, o dendrograma com o mesmo método de agrupamento anterior está apresentado na Figura 6. De forma análoga à Figura 4, pode-se perceber na linha vertical correspondente à distância igual a 5 que o dendrograma forma quatro grupos: G_1_ = fase 1, G_2_ = fase 3, G_3_ = fase 5 e G_4_ = fases 2 e 4. A fase 5 apresenta o maior índice de qualidade, evidenciando o fato de que se atingiu grau de maior qualidade.

**Figura 5:** Curvas da qualidade de execução das anastomoses (escore) de cada indivíduo ao longo das cinco fases do estudo.

**Figura 6:** Dendrograma pelo método hierárquico entre grupos pela distância euclidiana em percentis referente ao escore de qualidade observado nas fases.

Quando a árvore do dendrograma da Figura 4 é girada 90° graus à esquerda, um corte horizontal ilustrado pelas linhas (azul, laranja e verde) tracejadas em cada ponto resultante do tempo de cada fase é traçado, como demonstrado na Figura 7. Assim, observamos que, em G_1_ (fase 1), o tempo apresentado é consideravelmente maior que o tempo nas demais fases. No corte em G_2_ (fases 2 e 3), notamos uma diferença mínima entre o tempo de realização das anastomoses das fases 2 e 3. Já no G_3_ (horizontal fase 4 e 5), pela análise de agrupamentos, não há diferença significativa entre o tempo das anastomoses das fases 4 e 5. Isso sugere que, se o experimento fosse finalizado na fase 4, não haveria prejuízo na análise do tempo de realização das anastomoses.

**Figura 7:** Dendrograma entre grupos pela distância euclidiana em percentis referente aos tempos observados nas fases 1 a 5 (modificado 90° à esquerda).

Pode-se observar na Tabela 2 que há uma tendência crescente do índice de qualidade ao longo das fases e que não se detecta diferença estatística segundo o teste de Friedman apropriado para dados com nível de mensuração ordinal (escala de 1 a 5 na avaliação da qualidade). Portanto, não se detecta diferença pelo método estatístico, porém se percebe diferença clínica com a medida estatística mediana e com a análise de agrupamento. É provável que, em uma amostra de maior tamanho, se detectaria a diferença estatística pelo teste de hipótese de Friedman entre as fases.

**Tabela 2:** Medidas descritivas e sua comparação com o índice de qualidade ao longo das fases.

**Fase**	**Média**	**DP**	**Mediana**	**Valor - p**
1	2,50	1,049	2,50	0,603
2	3,00	0,632	3,00	
3	3,00	1,549	3,00	
4	2,83	0,753	3,00	
5	3,33	1,366	3,50	

DP: desvio-padrão.

## DISCUSSÃO

A técnica cirúrgica pode ser definida como o conjunto de manobras manuais ou instrumentais executadas pela equipe cirúrgica para a realização de um ato operatório. Assim, o conhecimento fundamental em técnica cirúrgica inclui o manuseio de instrumentos e a manipulação geral dos tecidos, a fim de se executar manobras comuns a todos os procedimentos cirúrgicos^17^^,^^18^. Dessa forma, desde a graduação até o período de residência médica, o desenvolvimento de habilidades manuais requer treinamento prático contínuo.

As anastomoses vasculares são importantes não só na cirurgia vascular, mas na prática cirúrgica em geral, como na revascularização miocárdica e cerebral, no trauma, nos transplantes, na cirurgia plástica e no reparo de lesões iatrogênicas, sendo seu ensino essencial nos programas de residência médica^19^^-^21. Anteriormente, os residentes adquiriam habilidades técnicas diretamente no bloco cirúrgico, sob supervisão de um cirurgião assistente. No entanto, nos últimos anos, diversos trabalhos têm apresentado propostas de treinamento para que o futuro cirurgião tenha contato com as anastomoses vasculares previamente^4^.

Nesse sentido, o modelo animal é muito citado na literatura e apresenta como uma das vantagens a sua proximidade em relação às características fisiológicas, anatômicas e orgânicas do ser humano^22^. Achar et al.^2^ desenvolveram um modelo com traqueia e esôfago de frango para o treinamento de anastomose término-terminal. Já Garbin et al.^23^ compararam o aprendizado de alunos após o treinamento de anastomose arteriovenosa látero-lateral, em modelo que utilizava línguas bovinas. Contudo, além da limitação ética, esse tipo de modelo também demanda um ambiente adequado para o preparo e a conservação do material.

Em contrapartida, a indústria desenvolveu simuladores artificiais para treinamento em anastomoses vasculares, porém o elevado custo dificulta o acesso por grande parte das instituições do Brasil. Isso levou ao desenvolvimento de simuladores alternativos como uma proposta mais acessível de modelo de treinamento^13^^-^15. No nosso estudo, a escolha pelo modelo de treinamento foi motivada justamente pela indisponibilidade de acesso aos modelos animais e aos simuladores disponíveis no mercado. Logo, de acordo com a realidade da nossa instituição, um modelo com material inerte, reprodutível e de baixo custo se mostrava o mais adequado.

Sabe-se que a simulação em modelos inanimados permite o treinamento repetitivo que leva a melhora da performance, garantindo segurança ao aluno sem a possibilidade de causar dano aos pacientes^23^^-^25. Atlan et al.^26^ desenvolveram um modelo sintético para treinamento em anastomoses microvasculares utilizando tubos de gelatina com álcool polivinílico em comparação com vasos de roedores, demonstrando uma melhora no desempenho e reduzindo o uso de animais.

O simulador proposto por Sarmento et al.^15^ se mostrou viável e de baixo custo, porém ainda não havia sido testado por alunos de graduação. De acordo com esse trabalho, a prática de anastomoses com o simulador permitiria a familiarização com o instrumental vascular específico e com o uso de fios de pequeno calibre e com duas agulhas, melhorando a destreza e a agilidade da técnica com movimentos delicados. Nesta perspectiva, confrontamos o modelo desenvolvido a partir da criação de uma curva de aprendizado.

As curvas de aprendizado são muito utilizadas, pois permitem avaliar a evolução do indivíduo ao longo de repetições de uma mesma técnica. Em geral, os parâmetros mais utilizados são o tempo e a qualidade do procedimento^27^. Um estudo com residentes de radiologia utilizando o treinamento com simulação em punção de articulações demonstrou melhora no tempo e na qualidade^28^. Yoshida et al.^11^ também descreveram o decréscimo no tempo em um estudo feito com cirurgiões vasculares.

É importante salientar que a redução do tempo de realização de um procedimento por si só não é suficiente para que determinada habilidade seja aprimorada. É necessário que haja também uma melhora dos parâmetros técnicos que predizem a qualidade do procedimento a fim de diminuir as complicações perioperatórias. Um estudo com residentes de cirurgia geral relacionou a competência em modelo laboratorial com a competência cirúrgica, analisando não apenas o tempo de conclusão da anastomose, mas também o grau de vazamento anastomótico e o número de vazamentos^29^.

O escore mais citado na literatura para avaliação do desempenho em anastomoses vasculares é o *Objective Structured Assessment of Technical Skills* (OSATS), que permite avaliar as habilidades cirúrgicas de maneira confiável e válida. Esse método envolve a observação direta de residentes executando uma variedade de tarefas operacionais estruturadas^30^. Assim, pela necessidade de examinador ou de gravação em todas as fases, optou-se pela utilização de um escore personalizado que permitisse a avaliação somente ao término de cada anastomose.

No presente estudo, observou-se uma redução no tempo de todos os alunos, com diferença estatística da fase 1 quando comparada com as fases 4 e 5. A análise descritiva mostrou uma tendência ao aumento de qualidade das anastomoses ao longo das fases, através da diferença clínica com a medida estatística mediana e com a análise de agrupamento.

No entanto, não foi detectada diferença pelo método estatístico no decorrer das fases. Isso pode ser justificado pela necessidade de grande número de repetições para alcançar uma boa qualidade. Um estudo feito com 15 cirurgiões, no qual realizaram cirurgia coronariana, demonstrou que são necessários 4 anos de prática para adquirir competência técnica e que o tempo de treinamento reduz progressivamente a mortalidade^31^. Assim, é provável que um estudo com maior número de fases ou amostra com maior número de alunos demonstre um resultado de melhora da qualidade com significância estatística.

Por outro lado, é importante citar que existe grande variação entre as habilidades manuais dos alunos. Sabe-se que o aprendizado em cirurgia é influenciado por uma complexa interação de fatores que incluem a capacidade inata do indivíduo, a experiência cirúrgica prévia e a motivação^32^. Comparando as curvas de aprendizado de estudantes em videolaparoscopia, observou-se importante grau de variabilidade intersubjetiva: enquanto alguns melhoraram gradualmente, outros melhoraram abruptamente^33^. Portanto, alguns indivíduos podem ter mais facilidade do que outros em aprender determinado procedimento, o que também difere no tempo necessário para que cada um alcance a qualidade desejada. Além disso, alcançar uma qualidade perfeita não foi o objetivo principal do nosso estudo, mas sim auxiliar no aprendizado da técnica cirúrgica.

Dessa forma, o modelo alternativo com balões de látex introduziu os discentes no assunto sobre a técnica de anastomose vascular, sendo uma ferramenta eficaz para melhorar o aprendizado global da técnica. Como perspectivas futuras, esperamos inserir o protótipo nos programas de residência médica de forma a ajudar no treinamento dos residentes e, por sua vez, reduzir os treinamentos em pacientes.
